# A new method for unmanned aerial vehicle path planning in complex environments

**DOI:** 10.1038/s41598-024-60051-4

**Published:** 2024-04-22

**Authors:** Yong He, Ticheng Hou, Mingran Wang

**Affiliations:** School of Electrical and Information Engineering, Changsha University of Science and Technology, Changsha, 410114 China

**Keywords:** Aerospace engineering, Mechanical engineering

## Abstract

To solve the problems of UAV path planning, such as low search efficiency, uneven path, and inability to adapt to unknown environments, this paper proposes A double-layer optimization A* and dynamic window method for UAV path planning. Firstly, the neighboring node clip-off rule is defined to optimize the node expansion mode of the A* algorithm, and the obstacle coverage model is designed to dynamically adjust the heurizing function of the A* algorithm to improve the path search efficiency. Then, the Bresenham algorithm is adopted for collision detection and critical path nodes are extracted to significantly reduce the number of path turning points. Secondly, a new tracking index is proposed to optimize the evaluation function of the dynamic window method to make the local path fit the global path further. By detecting the dangerous distance, the dynamic adaptive method of evaluation function weight is designed to improve the fixed weight of the dynamic window method. Finally, the key turning point of optimizing the A* algorithm is taken as the temporary target point to improve the DWA algorithm, and the local part follows the global part, and the fusion of the two algorithms is realized. Simulation results show that the proposed method can significantly improve the efficiency and smoothness of mobile robot path planning, enhance the real-time obstacle avoidance and adaptive ability of unknown environments, and better meet the requirements of complex planning tasks.

## Introduction

With the development of society, quadcopter drones are widely used in agriculture, power inspection, aerial imaging, national defense, and other fields^1–3^. This imposes higher requirements on the intelligent operational capabilities of drones. Depending on the different application scenarios of drones, path-planning algorithms for drones can be divided into static global algorithms and dynamic local algorithms.

Common global algorithms include graph-based search algorithms such as Dijkstra's algorithm^4^ and A* algorith^5,6^; sampling-based algorithms such as Rapidly-exploring Random Trees (RRT)^7^ and Probabilistic Roadmaps (PRM)^8^; intelligent algorithms such as reinforcement learning^9^, deep reinforcement learning^10^, etc. Static global path planning involves finding an optimal path for a drone in a known environment, with the A* algorithm widely applied due to its advantages in speed and optimal path generation^11^. However, it has issues like high memory consumption and poor path quality. Chen^12^ proposed an approximately optimal bidirectional A* search algorithm, which rapidly completes path planning by selecting optimal node pairs. Daniel^13^ introduced the Theta* and Angle-Propagation Theta* algorithms, both capable of planning smoother paths at arbitrary angles on grid maps. Hu Shiqiang^14^ combined vector cross-product with a scale balancing factor to optimize the A* heuristic function. They employed a jump-point search strategy, achieving variable step-size search. Wang Bin^15^ combined an improved A* algorithm with dynamically adjustable heuristic function weights and a dynamic window approach based on an environment-adaptive improvement strategy. This not only solves issues in traditional A* and Dynamic Window Approach like redundancy, low efficiency, and path redundancy but also enhances path safety and real-time performance, aligning better with the motion characteristics of mobile robots. Zhao Xiao^16^ improved A* with a jump point search algorithm, reducing unnecessary memory consumption. However, most A* and its improved algorithms lack obstacle avoidance capabilities, leading to planning failures when the environment changes. Wang Hongbin^17^ designed the Optimal Target-First Search method, which sorts multiple targets based on cost, and sequentially plans paths for each target point. Subsequently, the A* algorithm is employed for secondary optimization. Zhang Zhen^18^ incorporates environmental and parent node information into the A* evaluation function and designs a safe extension strategy to dynamically change the extension direction, thereby improving pathfinding efficiency and obstacle avoidance capability.

Local path planning algorithms include the dynamic window approach^19,20^, artificial potential field method^21^, etc. Local algorithms calculate progressively using the latest sensor information during motion and can effectively avoid both known and unknown obstacles. Li Xinying^22^ used an improved multi-objective particle swarm algorithm to dynamically adjust the weight coefficients of the Dynamic Window Approach. The adaptive weighting problem is transformed into a multi-objective optimization problem, and an improved particle swarm algorithm is employed for optimization, enabling the adapted DWA algorithm to dynamically adjust parameters based on the environment. Wang Yongxiong^23^ proposed a parameter-adaptive DWA algorithm that adjusts the weights in the objective function based on the distance between the robot and obstacles and the density of obstacles, resulting in more reasonable paths. Chou et al.^24^ improved the DWA algorithm by incorporating region analysis techniques. They filter incorrect commands through lookahead verification to guide the path toward optimal results. Chang^25^ defined state and action spaces, and through dynamically adaptive adjustments using reinforcement learning to the sub-evaluation functions of DWA, they improved the environmental adaptability and dynamic obstacle avoidance performance of the DWA algorithm.

The algorithms mentioned above have been improved in various aspects for A* and the Dynamic Window Approach, enhancing operational efficiency and environmental adaptability to some extent. However, issues such as path redundancy and high memory consumption persist. Therefore, this paper proposes a dual-layer optimization algorithm. Firstly, neighbor node pruning rules are defined, and obstacle coverage is dynamically adjusted to regulate the weight of the heuristic function, allowing it to adaptively adjust based on environmental changes, thereby improving planning efficiency. Secondly, the Bresenham algorithm is employed to extract key path nodes, and a deviation function is designed to enhance the followability of the DWA algorithm in local path planning, with simultaneous dynamic adjustments of evaluation function weights based on the environment. Finally, the extracted key path nodes are utilized as temporary sub-goals, endowing the A* algorithm with the ability to navigate around unknown obstacles. The effectiveness of the improved algorithm is verified through simulation and experimentation.

## Introduction to A* algorithm

A* algorithm is built upon Dijkstra's algorithm by incorporating a heuristic function to guide the search. It is capable of finding the shortest path from the starting point to the destination, possessing completeness and optimality^26^. By maintaining two sets, Open and Closed, the A* algorithm iteratively expands nodes with the minimum cost. The path planning concludes when the destination is added to the Closed set or the Open queue becomes empty. The calculation of the node cost is done by

1 $$f(n) = g(n) + h(n)$$

where,$f(n)$ represents the total cost from the starting point through the node to the target point; $g(n)$ represents the actual cost from the starting point to the node; $h(n)$ represents the heuristic estimated cost from the node to the target point. The $h(n)$ primarily has two calculation methods: Euclidean Distance and Manhattan Distance^27^, specified as follows:

2 $$h(n) = \sqrt {\left( {m_{x} - n_{x} } \right)^{2} + \left( {m_{y} - n_{y} } \right)^{2} }$$

3 $$h(n) = |m_{x} - n_{x} | + |m_{y} - n_{y} |$$

where, $(m_{x} ,m_{y} )$ is the current node coordinate, $(n_{x} ,n_{y} )$ is the target node coordinates, Due to the relatively complex and computationally intensive nature of calculating Euclidean Distance, this paper adopts the Manhattan Distance formula.

Although the A* algorithm can find the shortest path required to complete a task in a known environment, it has a broad search scope, low planning efficiency, and is unable to navigate around unknown obstacles.

## Dynamic window approach

The DWA algorithm possesses the advantages of efficiency, real-time capability, strong obstacle avoidance, and ease of implementation. It primarily performs discrete sampling within the allowed velocity space based on the current motion state of the drone and simulates the motion trajectories of these velocity combinations within the forward prediction time. Subsequently, the trajectory scores are determined by the evaluation function, leading to the identification of the optimal trajectory^28^. Subject to the constraints of motor performance and the environment, the velocity of the unmanned aerial vehicle at time t + 1, denoted must satisfy the following constraints:

1. Due to the constraints imposed by the hardware performance of the unmanned aerial vehicle, the velocity constraints for the drone are expressed as:
  4 $$V_{m} = \left\{ {v_{x} \in [v_{\min } ,v_{\max } ],v_{y} \in [v_{\min } ,v_{\max } ],\omega \in [\omega_{\min } ,\omega_{\max } ]} \right\}$$
  where, $v_{\max }$ and $v_{\min }$ represent the maximum and minimum linear velocity constraints; $\omega_{\max }$、$\omega_{\min }$ correspond to the maximum and minimum angular velocity constraints.
2. Subject to the constraints of the unmanned aerial vehicle's motor performance, the drone should ensure velocity vector space sampling within the range that the motor torque can withstand, namely the unmanned aerial vehicle motor acceleration constraints^29^:
  5 $$V_{d} = \left\{ {\begin{array}{*{20}c} {v_{x} \in [\max (v_{\min } ,v_{x} - a_{x\min } \Delta t),\min (v_{\max } ,v_{x} + a_{x\min } \Delta t)]} \\ {v_{y} \in [\max (v_{\min } ,v_{y} - a_{y\min } \Delta t),\min (v_{\max } ,v_{y} + a_{y\min } \Delta t)]} \\ {\omega \in [\max (\omega_{\min } ,\omega - a_{\omega \min } \Delta t),\min (\omega_{\max } ,\omega + a_{\omega \min } \Delta t)]} \\ \end{array} } \right.$$

Here, $a_{x\min } ,a_{x\max }$ represents the maximum deceleration and acceleration in the UAV’s x-axis direction. $a_{y\min } ,a_{y\max }$ represents the maximum deceleration and acceleration in the UAV’s y-axis direction. $a_{\omega \min } ,a_{\omega \max }$ represents the maximum deceleration and acceleration in the UAV's angular velocity.

The original evaluation function of the DWA algorithm is given by:

6 $$G(v,\omega ) = \sigma (\alpha \cdot heading(v,\omega ) + \beta \cdot dist(v,\omega ) + \gamma \cdot vel(v,\omega ))$$

where,$heading(v,\omega )$ is the heading sub-function, used to evaluate the extent to which the trajectory aligns with the target. $dist(v,\omega )$ is the distance sub-function, predicting that the farther the trajectory is from obstacles, the higher the score for this term. $vel(v,\omega )$ is the velocity sub-function, characterizing the swiftness of the flight. $\alpha ,\beta ,\gamma$ is the weight coefficient for the respective terms, $\delta$ represents the normalization process.

## Adjacency node clipping rule

**Figure 1:**
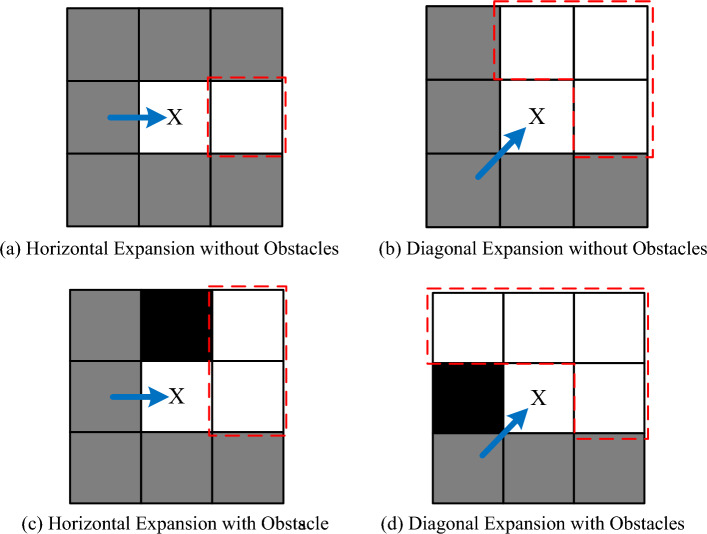
Adjacency node clipping rule. (**a**) Horizontal expansion without obstacles, (**b**) diagonal expansion without obstacles, (**c**) horizontal expansion with obstacles, (**d**) diagonal expansion with obstacle.

Based on the expansion direction between the parent node and the current node, the neighbor node pruning rules are defined, as shown in Fig. 1. Here, gray grids represent free nodes that are unoccupied by obstacles and do not need to be considered. White grids represent neighboring nodes that the current node needs to consider, and black grids represent obstacles. The specific neighbor node pruning rules are as follows:

In the absence of obstacles, if the current node is expanded from the parent node in the horizontal or vertical direction, then the current node's neighbors only need to consider one node in the same expansion direction, as shown in Fig. 1a. Other neighboring nodes can be reached from the parent node without passing through the current node at a lower cost, eliminating the need for expansion. In the case of diagonal expansion, as depicted in Fig. 1b, the situation is similar to straight-line expansion, with the only difference being that paths not passing through the current node must strictly dominate, facilitating subsequent recursive searches. When there are obstacles around the current node, and the parent node does not have a better path to reach certain nodes without passing through the current node, such neighboring nodes also need to be considered, as illustrated in Fig. 1c and 1d.

## Adaptive A* heuristic function

The A* algorithm balances accuracy and speed using a heuristic function. However, the heuristic function often underestimates the distance from the current node to the goal, resulting in low search efficiency. To address this, this paper introduces obstacle coverage rate to abstract environmental information. It constructs a function similar to a sigmoid function to dynamically adjust the weight of the heuristic function, aiming to enhance the efficiency of path planning. The main idea is as follows: when there are many obstacles in the environment, increase the heuristic function value of nodes appropriately to reduce the error between the estimated distance and the actual distance, thereby improving search speed. Conversely, when there are fewer obstacles, the estimated cost between nodes and the goal will be closer to the actual value. In this case, appropriately decrease the magnitude of weight adjustment to better balance search accuracy and speed. The new cost function and obstacle coverage rate are defined as follows:

7 $$f(n) = g(n) + (1 + \frac{1}{{1 + e^{ - \xi } }})h(n)$$

8 $$\xi = \frac{{\sum\limits_{{i = n_{x} }}^{{g_{x} }} {\sum\limits_{{j = n_{y} }}^{{g_{y} }} {value(i,j)} } }}{{\left| {n_{x} - g_{x} } \right|*\left| {n_{y} - g_{y} } \right|}}$$

9 $$value(i,j) = \left\{ \begin{gathered} 1,if(i,j) \subseteq C_{obs} \hfill \\ 0,if(i,j) \subseteq C_{free} \hfill \\ \end{gathered} \right.$$

Here, $C_{obs}$ and $C_{free}$ respectively represent obstacle space and free space; $value(i,j)$ is the value function for coordinates $(i,j)$, indicating whether the corresponding grid is occupied by an obstacle.

## Bresenham algorithm extracts key nodes

**Figure 2:**
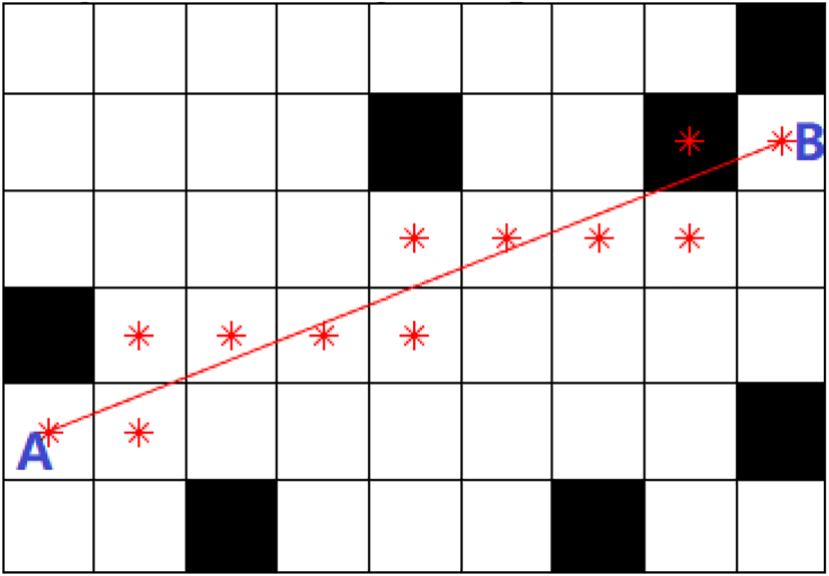
Bresenham algorithm for collision detection.

When using the A* algorithm for path planning, the planned path often contains redundant and turning points, increasing memory overhead, and the path is not smooth. Further optimization is required. In this paper, a strategy for extracting key path nodes is proposed based on the direction of node expansion and the Bresenham algorithm. The Bresenham algorithm is a linear scan conversion method^30^ that can determine the grid area through which the line connecting two nodes passes quickly by using the slope and intercept of the line. This facilitates the collision detection process. As shown in Fig. 2, the grid area through which the line connecting nodes A and B passes has obstacles, indicating a collision in that path segment, making it impassable.

The steps for extracting critical path nodes are as follows:

1. Differentiating Redundant Nodes and Turning Points Based on Node Expansion Direction: Define $Q\left( {i,i + 1} \right)$ as the expansion direction between two adjacent nodes ${P}_{i}$ and ${P}_{i+1}$. If $Q\left( {i - 1,i} \right)$ is the same as $Q\left( {i,i + 1} \right)$, then node ${P}_{i}$ is considered a redundant node; otherwise, it is classified as a turning point.
2. Using the Bresenham algorithm to identify key path nodes: Starting from the initial point ${P}_{1}$, connect to the next turning point $P_{j}$ and subsequent nodes $P_{j + k}$.Conduct collision detection sequentially. If the first collision occurs on the line connecting node ${P}_{1}$ to $P_{j + k}$,then node $P_{j + k - 1}$ is considered a key path point.
3. Starting from the new key path node, repeat step (2) until the target point is extracted.

## Improving the dynamic window approach

By utilizing the key path nodes extracted through the A* algorithm as intermediate target points for the Dynamic Window Approach, coupled with the real-time obstacle avoidance capability of the Dynamic Window Approach, the improved A* algorithm can promptly respond to and navigate around unknown obstacles in the planning environment. This enhances the environmental adaptability and success rate of A* algorithm path planning. Considering the tracking performance of local path planning after obstacle avoidance, this paper introduces the global path-following subfunction to improve the local path-following effect and achieve global optimization. The evaluation function and distance sub-function calculation method for improving the Dynamic Window Approach algorithm are as follows:

10 $$\left\{ {\begin{array}{*{20}l} {G(v,w) = \delta [\alpha \cdot heading(v,w) + \, \beta \cdot vel(v,w) + Dist(v,w)]} \hfill \\ \begin{gathered} Dist(v,w) = \lambda \cdot dist_{obs} (v,w) + \eta \cdot dist_{fol} (v,w) \hfill \\ heading(v,w) = \pi - \Delta \theta \hfill \\ \end{gathered} \hfill \\ \end{array} } \right.$$

where,$heading(v,w)$ is the azimuth evaluation function, which measures the error $\Delta \theta$ of the Angle between the position direction of the end point of the trajectory and the line of the target point generated at the current sampling speed;$Dist(v,w)$ is the distance evaluation function, $dist_{obs} (v,w)$ is the obstacle avoidance function, representing the minimum distance between the end of the current trajectory and the obstacle. It is used to punish the sampled trajectory near the obstacle to ensure the obstacle avoidance ability of the drone. The closer the distance, the lower the score. $dist_{fol} (v,w)$ is the following function, indicating the distance between the global optimal path and the current trajectory. The higher the trajectory score is, the better the trajectory is. The DWA algorithm will select the sampled trajectory action with the highest score.

In complex and dynamic environments, the Dynamic Window Approach with fixed weight values may encounter planning failures or result in unreasonable paths^31^. This paper proposes a method with adaptive weightings, selecting weight combinations based on the distance between the robot's current position and obstacles. This allows the robot to generate more reasonable scores in diverse environments. The specific steps are as follows:

- Define the detection distance between the current position and obstacles as $dist_{now}$, The warning distance between UAV and obstacle is $dist_{alert} = 2\;{\text{m}}$, and the danger distance as $dist_{risk} = 1.5\;{\text{m}}$.
- When $dist_{now} \le dist_{alert}$,indicates that the robot is close to the obstacle, and a response to enter the safe zone is required. At this point, it is necessary to increase $\lambda$ which is the weight value of the obstacle avoidance function $dist_{obs} (v,w)$ and decrease $\eta$ which is the weight value of the following function $dist_{fol} (v,w)$.
- When $dist_{now} \le dist_{risk}$,indicating that the current distance is within the danger distance, the primary task is to leave this area. Therefore, it is necessary to continue increasing the obstacle avoidance weight.
- When $dist_{now} > dist_{alert}$,indicating that the robot is in a safe zone, in order to improve local path-following performance, it is necessary to increase the weight value of the following function $dist_{fol} (v,w)$ and decrease the weight value of the obstacle avoidance function $dist_{obs} (v,w)$. The weight combination is as follows:

## UAV performance analysis

UAV path planning involves the pre-flight formulation of an optimal reference trajectory that satisfies constraints based on environmental information and mission requirements. During flight, in the presence of unknown or dynamic threat information, local trajectory optimization is performed dynamically. The global planning objective is to avoid convergence to local optima and minimize computational complexity, while local optimization focuses on reducing planning time for real-time responsiveness. In certain tasks, UAVs may neglect changes in altitude by projecting three-dimensional objects onto a two-dimensional plane to simplify problem complexity and enhance trajectory planning efficiency. Two-dimensional path planning finds extensive applications across various domains including agricultural crop protection, search and rescue operations, and aerial photography, among others.

When engaging in path planning for UAVs, it is crucial to comprehensively account for the constraints arising from the UAV's inherent performance limitations, thereby ensuring the seamless execution of tasks. The constraints integral to UAV path planning encompass factors such as the maximum turning angle and the maximum trajectory length, both of which must be adhered to for effective planning.

Maximum Turning Angle: During UAV flight, the turning angle is limited by the aircraft's performance, and it must adhere to a specific range in order to determine the agility of the UAV. If $(x_{i} ,y_{i} )$ represents the current position of the UAV in segment $i$, and $\Delta \theta_{i}$ denotes the required turning angle for transitioning to the next segment,$\theta_{\max }$ represents the maximum allowable turning angle for optimal UAV performance, satisfying:

11 $$\Delta \theta_{i} < \theta_{{_{\max } }}$$

Maximum track length: The UAV needs to avoid obstacles during flight, and it is difficult to maintain a straight line. The maximum flight path of the UAV is affected by its own battery energy consumption, so the maximum flight path of the UAV will be constrained. If the track path is composed of several sections $$l_{i}$$$$(i = 1,2, \cdots ,n)$$ and the maximum track length of the UAV is $$L_{\max }$$, then:

12 $$\sum\limits_{i = 0}^{n} {l_{i} } < L_{\max }$$

## Fusion algorithm

The advantage of the A* algorithm lies in its ability to plan the shortest path in an environment with static obstacles. However, the planned path often contains many turning points and redundant nodes. Moreover, in dynamic environments, the path generated by A* in a static environment may not effectively avoid obstacles.

**Figure 3:**
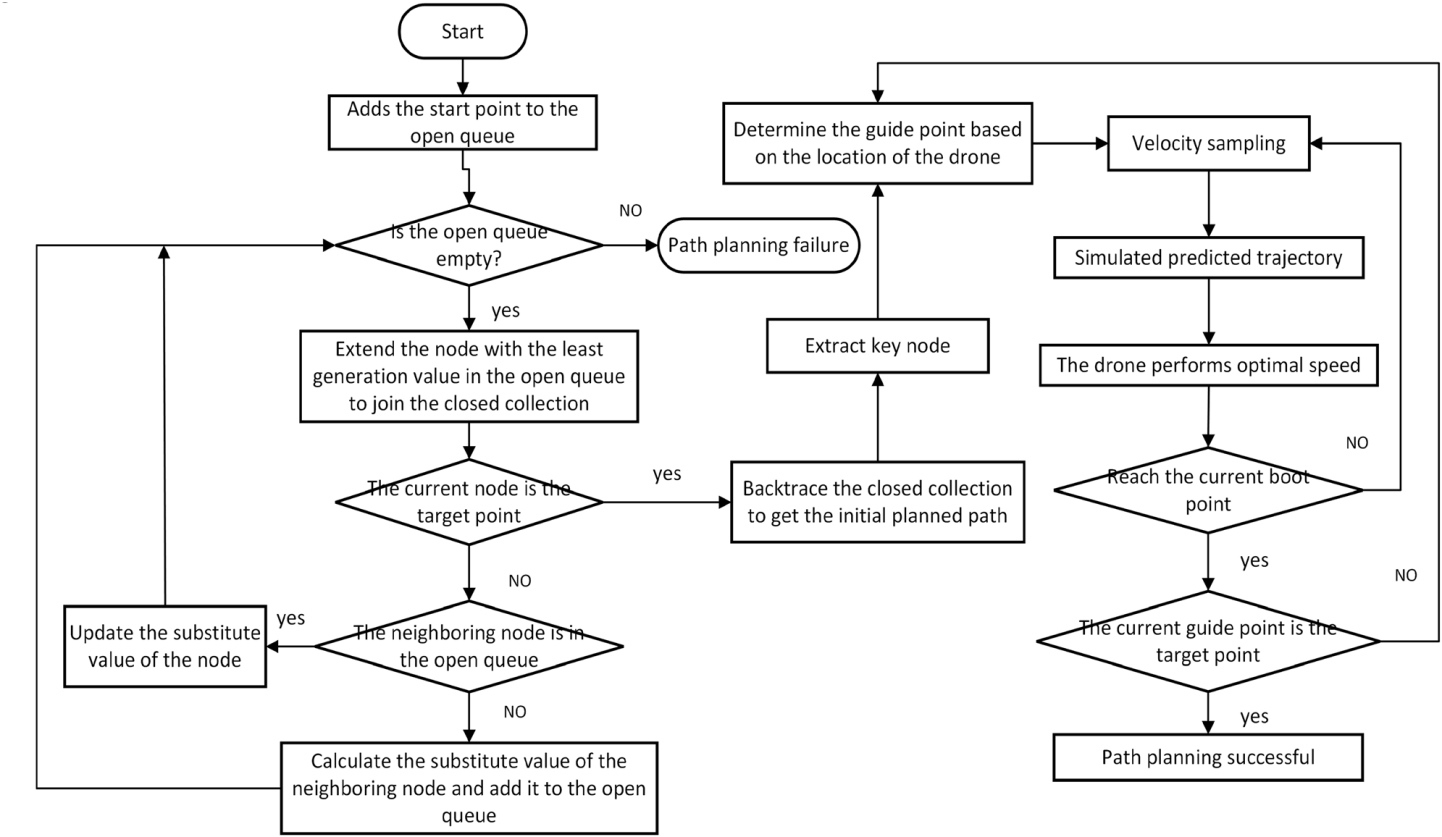
Flow chart of fusion algorithm.

Due to the lack of guidance from a global path, the traditional Dynamic Window Approach algorithm often struggles to obtain an ideal optimal path, especially in complex obstacle environments, leading to a tendency to get stuck in local optima and be unable to reach the target point. Therefore, this paper integrates the global path planning information from the improved A* algorithm to guide the DWA algorithm. This ensures local dynamic obstacle avoidance while achieving global path optimality. In the end, the workflow of the integrated algorithm is depicted in Fig. 3.

## Fusion algorithm simulation analysis

To validate the effectiveness and superiority of the improved A* algorithm, a simulation-based comparative analysis was conducted among the A* algorithm, Theta* algorithm, and the improved algorithm proposed in this paper using different environmental maps. The computer configuration includes a Windows 10 64-bit operating system, an Intel (R) Core (TM) i5-10210U CPU with a base frequency of 1.60 GHz (turbo boost up to 2.11 GHz), and 16 GB of RAM. MATLAB R2017b was used as the simulation software, and the parameter values used in the simulation are detailed in Table 1, where, $v_{\max }$, $\omega_{\max }$ are the maximum linear velocity and maximum angular velocity of local path planning, respectively, and $a_{v \cdot \max }$, $a_{\omega \cdot \max }$ are the maximum linear acceleration and maximum angular acceleration, respectively, and $R$ is the sensing range radius of the UAV.

**Table 1:** Simulation parameters and values.

Parameter	Value	Parameter	Value
$v_{\max }$ (m/s)	1	$a_{v \cdot \max }$(m/s^2^)	2
$\omega_{\max }$ (rad/s)	0.7854	$a_{\omega \cdot \max }$(rad/s^2^)	2.6180
$\Delta t$ (s)	0.1	$T$(s)	3
$R$ (m)	12 ~ 16	$N$	3

**Figure 4:**
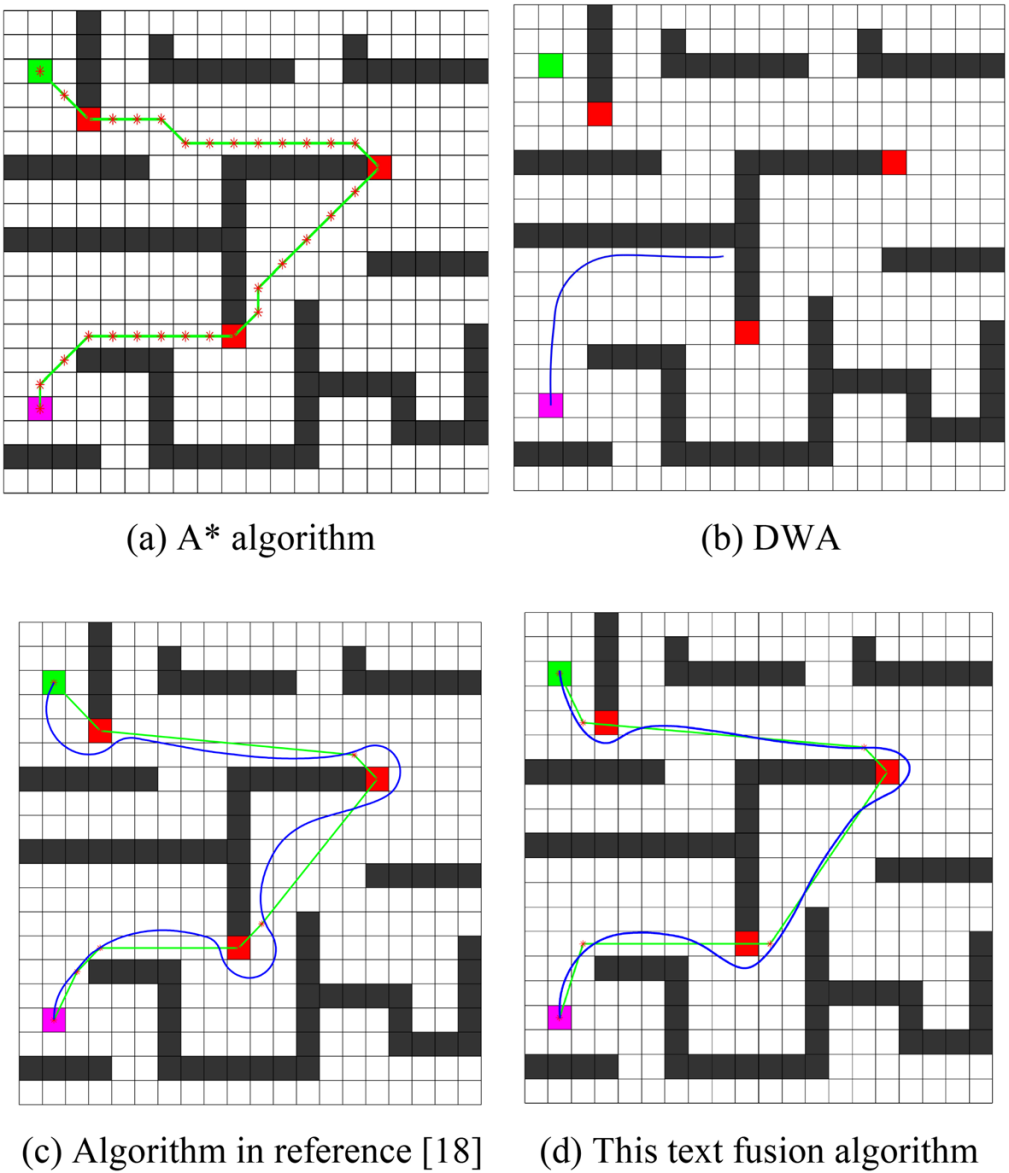
20 × 20 ladder obstacle map. (**a**) A* algorithm, (**b**) DWA, (**c**) Algorithm in reference^18^, (**d**) This text fusion algorithm.

**Figure 5:**
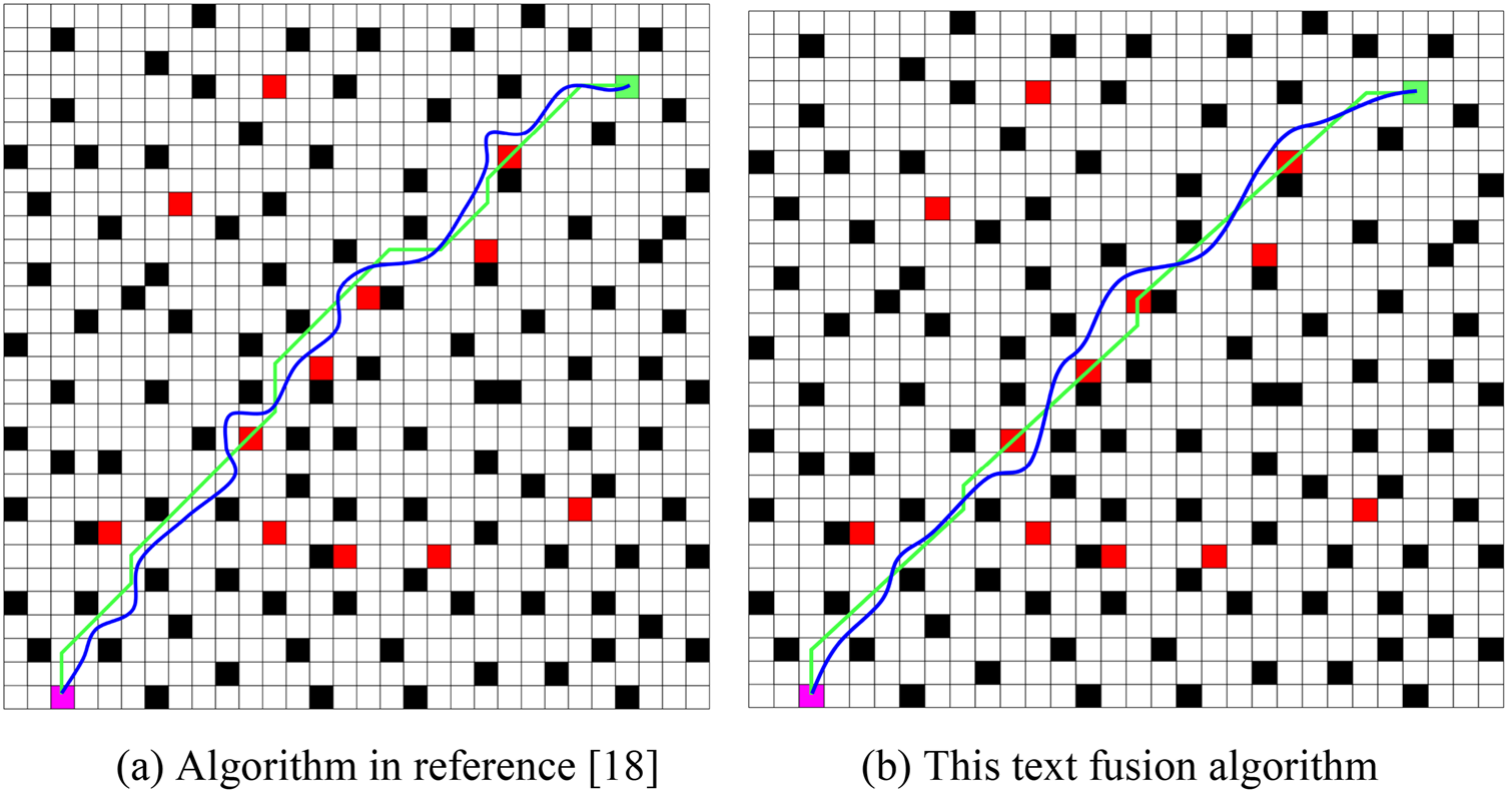
30 × 30 random obstacle map. (**a**) Algorithm in reference^18^, (**b**) This text fusion algorithm.

To validate the superiority of the algorithm proposed in this paper, simulations were conducted on grid maps with unknown obstacles of sizes 20 × 20 and 30 × 30. Traditional A*, traditional DWA algorithm, the algorithm proposed in^18^, and the fused algorithm proposed in this paper were compared. The experimental data is recorded in Table 2, and the experimental results are illustrated in Figs. 4 and 5. In these figures, magenta grids represent the starting points, green grids represent the target points, black grids represent known obstacles, red grid indicates unknown obstacles, green broken lines indicate global paths, blue curves indicate local paths, and nodes along the paths are marked with asterisks (*).

**Table 2:** Performance comparison of global path planning algorithms.

Map type	Algorithm	Run time/s	Number of extended grids /(PCS)	Total degree of corner /(°)	Whether smooth or not	Collision or not	Path length to target point/(m)
Ladder obstacle map	Traditional A*	–	–	–	Not	Yes	non-arrival
	DWA	60.12	–	–	Yes	Not	54.92
	Algorithm in reference^18^	56.58	186	349.3	Yes	Not	37.41
	Textual algorithm	23.44	151	294.6	Yes	Not	26.55
Random obstacle map	Algorithm in reference^18^	76.33	272	476.2	Yes	Not	52.32
	Textual algorithm	46.47	231	376.6	Yes	Not	46.68

From the above simulation results, it is evident that in Fig. 4a, the traditional A* algorithm fails to navigate around unknown obstacles, resulting in path planning failure. In Fig. 4b, the traditional DWA algorithm easily gets trapped in local optima, especially when faced with "U"-shaped obstacles, making it unable to reach the target point. Figure 4c shows that the algorithm proposed in^18^ successfully reaches the target point through dual-layer path planning; however, it exhibits large turning angles and poor local path following, still suffering from the issue of lengthy paths.In contrast, in Fig. 4d, the fused algorithm proposed in this paper successfully avoids unknown obstacles, resulting in smoother paths and stronger environmental adaptability. As indicated in Table 2, the runtime of the fused algorithm proposed in this paper is reduced by 58.6% and 61.1% compared to the algorithm in the literature and the DWA algorithm, respectively. Furthermore, the path length is reduced by 29.1% and 51.7%, with smaller turning angles, making it more suitable for UAV flight conditions.

To further validate the superiority of the fused algorithm proposed in this paper, comparative experiments were conducted with the algorithm in the literature on a 30 × 30 random obstacle map, as shown in Fig. 5.

From Fig. 5a and b, it can be observed that both the algorithm proposed in^18^ and the fused algorithm proposed in this paper successfully find a path from the starting point to the destination in an environment containing unknown and random obstacles. However, as indicated in Table 2, compared to the algorithm in the literature, the fused algorithm proposed in this paper reduces the number of expanded grids by 15.1%, decreases the runtime by 39.1%, and shortens the path length by 10.8%. The proposed algorithm exhibits significantly improved operational speed and planning efficiency, with better local path following characteristics. Its curvature is continuous, and its turning angles are smaller.

**Figure 6:**
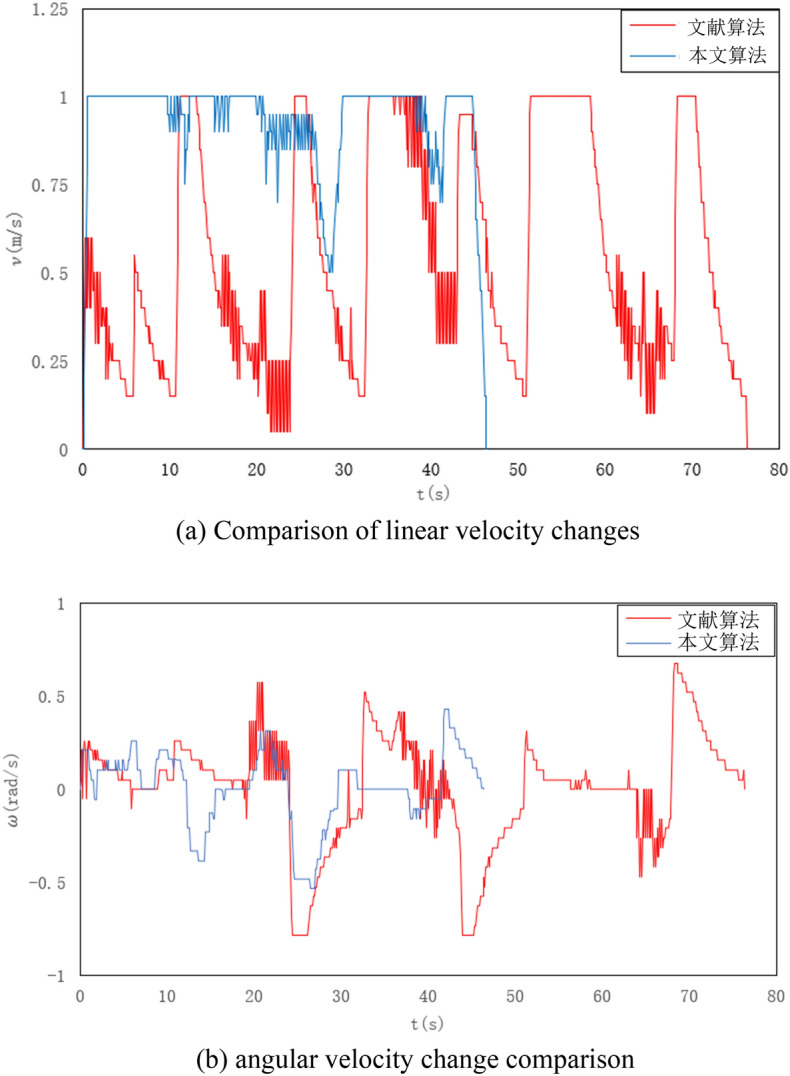
Comparison of speed changes in a 30 × 30 random obstacle map. (**a**) Comparison of linear velocity changes, (**b**) angular velocity change comparison.

Figure 6 shows the comparison between the linear velocity and angular velocity of the two algorithms in the path-planning process of a 30 × 30 random obstacle map. The average data records of multiple measurements are shown in Table 3. The simulation results reveal that the proposed algorithm exhibits small and gradual changes in linear and angular velocities when avoiding unknown obstacles. Compared to the literature algorithm, the proposed algorithm shows an average increase of 67.3% in linear velocity and a 33.3% improvement in average angular velocity, indicating a significant enhancement in algorithm speed.

**Table 3:** Comparison of algorithm performance indicators.

Algorithm	Mean linear velocity (m/s)	Mean angular velocity (rad/s)	Planning time (s)
Document algorithm	0.55	0.21	76.33
Textual algorithm	0.92	0.14	46.47

## Experimental research

**Figure 7:**
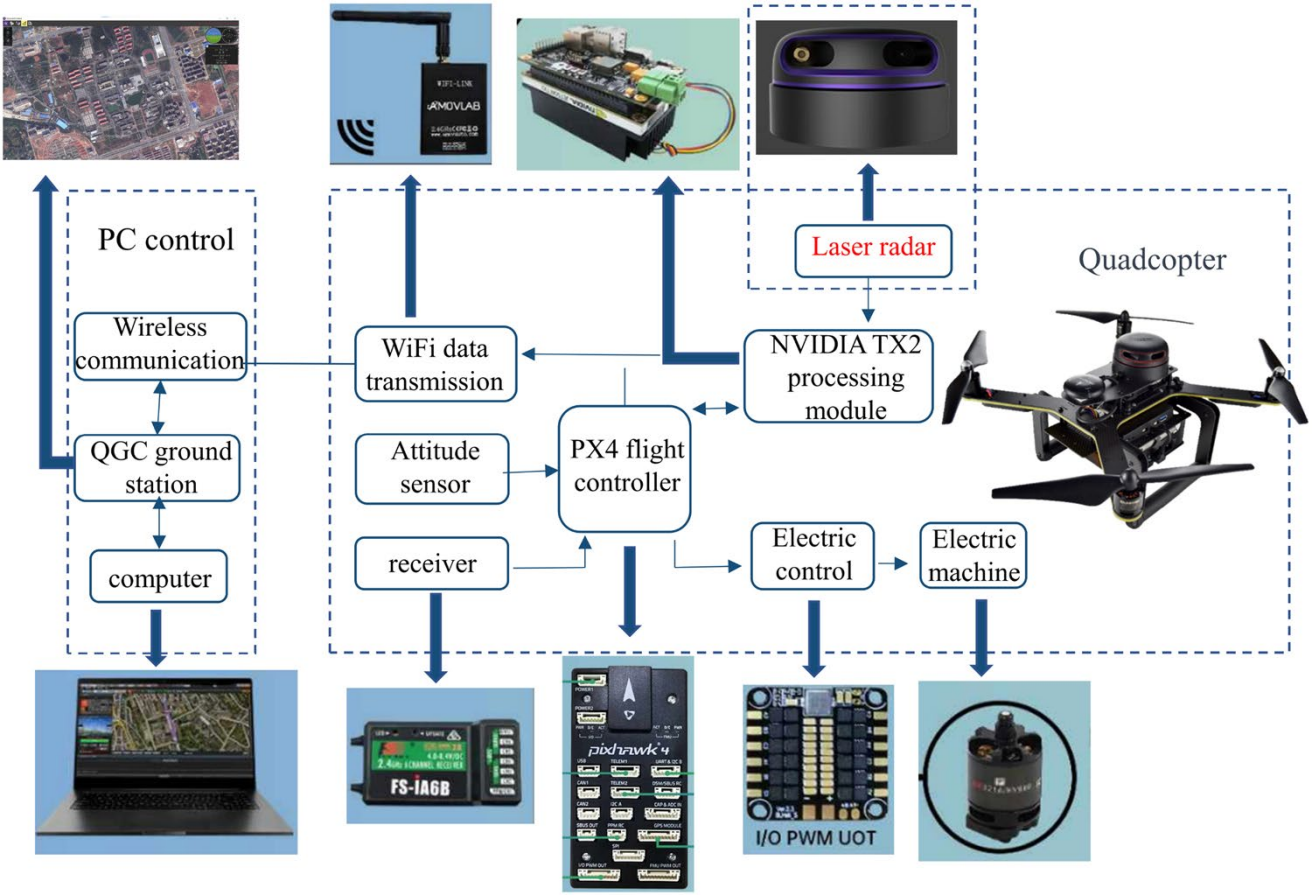
UAV experiment platform.

**Figure 8:**
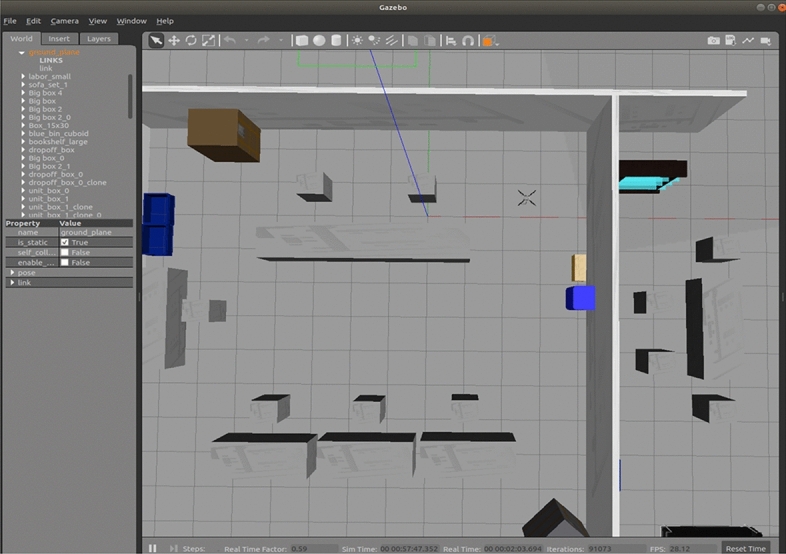
The experimental site in Gazebo is reproduced.

To further validate the effectiveness of the improved A* algorithm in practical applications, real-world experiments were conducted using the P200 unmanned aerial vehicle equipped with a SLAMTEC A2 LiDAR and Jetson TX2 onboard computer. The experimental UAV platform is illustrated in Fig. 7, and the experimental parameters are provided in Table 4. The onboard computer runs on Ubuntu 18.04, and the robot operating system is Melodic. Prior to the experiment, the gmapping algorithm was utilized for mapping, and the experimental results were visualized using the Rviz tool, as shown in Fig. 7. Figure 8 is a map of the experimental site reproduction in Gazebo.

**Table 4:** Weight combination of evaluation function.

Evaluation function weight	$\alpha$	$\beta$	$\lambda$	$\eta$
Following weight	0.3	0.3	0.2	0.2
Obstacle avoidance weight 1	0.2	0.3	0.4	0.1
Obstacle avoidance weight 2	0.2	0.25	0.5	0.05

**Figure 9:**
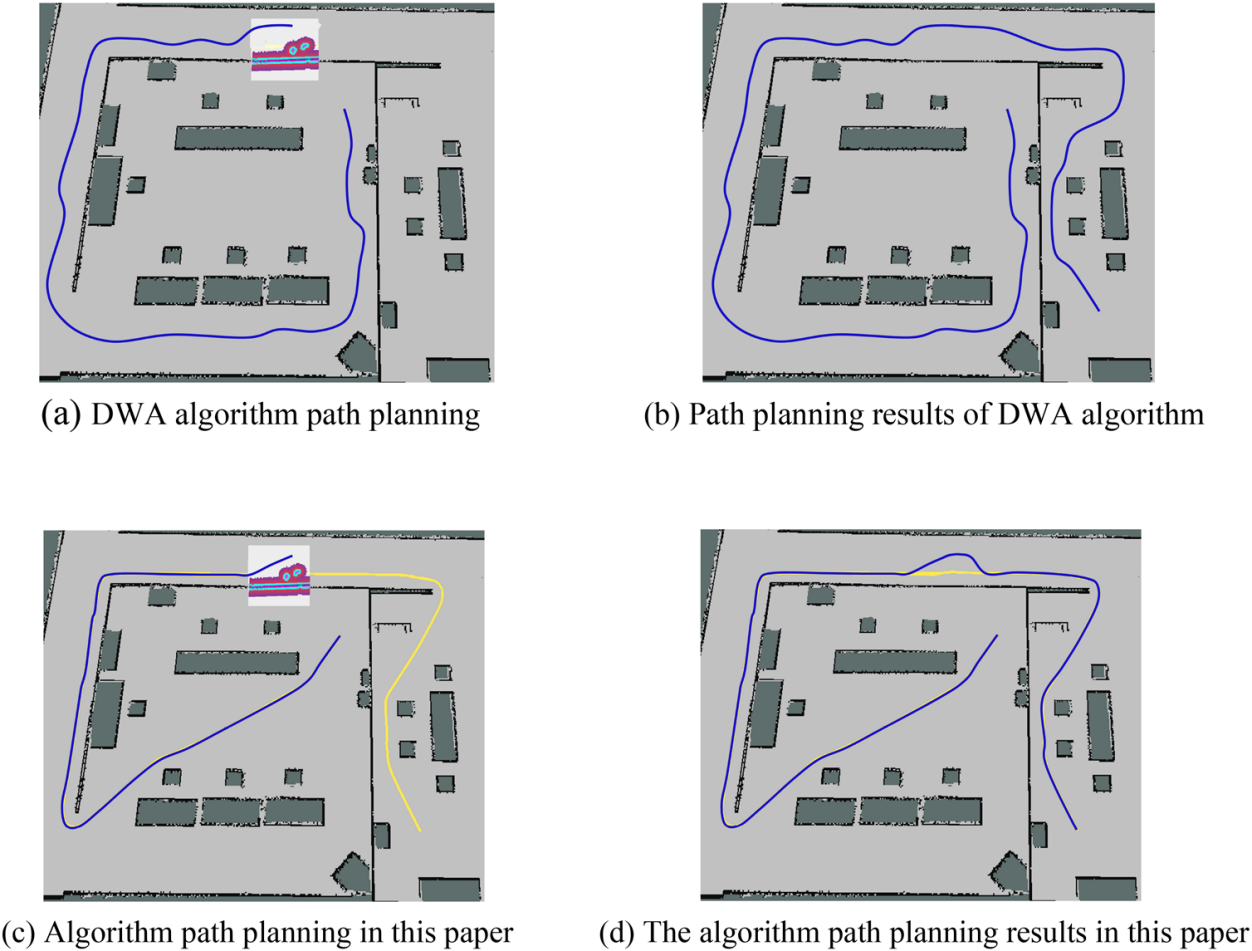
The comparison between the proposed algorithm and the DWA algorithm. (**a**) DWA algorithm path planning, (**b**) Path planning results of DWA algorithm, (**c**) Algorithm path planning in this paper, (**d**) The algorithm path planning results in this paper.

In Fig. 9, the yellow curve is the path planned by the improved A* algorithm on a static known map, which cannot avoid unknown obstacles but can provide global guidance. In Fig. 9a and c, the UAV senses the surrounding environment in real-time through the LiDAR and starts to move under the guidance of the planned path, where the white box is the set radar detection range, the colored area is part of the detected obstacles, and the blue curve is the actual flight path.

As can be seen from Fig. 9, compared with the traditional dynamic window method, the two-layer optimization algorithm proposed in this paper has a significantly shorter path. In addition to avoiding unknown obstacles, local planning has a better tracking ability for the globally optimal path, making the path smoother and the pathfinding efficiency greatly improved. It can be seen from Table 5 that in the same environment, the path length and pathfinding time of the proposed algorithm are reduced by 24.3% and 18.4% compared with the DWA algorithm. Therefore, in practical applications, the proposed algorithm has a short path length, high quality, stronger adaptability to the environment, and can avoid unknown obstacles on the path, and complete the path-finding task more efficiently.

**Table 5:** Comparison of experimental results of path planning.

Algorithm	Path length/(m)	Wayfinding time/(s)
Traditional DWA	56.35	67.31
Textual algorithm	56.35	54.92

## Conclusion

In order to effectively solve the problems of UAV path planning such as low search efficiency, uneven path, and inability to adapt to unknown environments, this paper proposes A double-layer optimization improved A* and dynamic window method for UAV path planning. Firstly, by designing the neighbor node clipping rule and defining the obstacle coverage rate, the heurizing function of the A* algorithm is introduced to dynamically adjust the environment information, so as to optimize the traditional node expansion mode and improve the path search efficiency. Secondly, the Bresenham algorithm is used for obstacle collision detection to extract critical path nodes, which effectively reduces path redundancy and improves path smoothness. Then, a following subfunction index is proposed to improve the ability of local planning to follow the global path. By judging the dangerous distance from obstacles in real-time, an adaptive method of evaluation function weight is designed to solve the problem of unreasonable path planning caused by the fixed weight of the DWA algorithm. Finally, the optimized key node is used as the temporary target point of local path planning, the local plan is guided to follow the global path, and the two-layer path planning is realized. The simulation and experimental results show that the proposed algorithm makes UAV path planning efficient and short in complex environments, and the smooth path is more suitable for UAV flight conditions. In the face of unknown obstacles, it can flexibly transform to avoid obstacles in real-time, which verifies the effectiveness and superiority of the proposed algorithm.

## Data Availability

The data that support the findings of this study are available from the corresponding author upon reasonable request.
